# The role of the adrenalectomy in the management of pheochromocytoma: the experience of a Portuguese referral center

**DOI:** 10.1007/s12020-024-03916-y

**Published:** 2024-06-08

**Authors:** Inês Costa Carvalho, Miguel V. B. Machado, João P. Morais, Filipa Carvalho, Elisabete Barbosa, José Barbosa

**Affiliations:** 1 Faculty of Medicine, University of Porto, Porto, Portugal; 2 Department of General Surgery, Centro Hospitalar Universitário de São João, Porto, Portugal; 3 Genetics, Department of Pathology, Faculty of Medicine, Porto, Portugal; 4 i3s—Instituto de Investigação e Inovação em Saúde, University of Porto, Porto, Portugal; 5 Department of Surgery and Physiology, Faculty of Medicine, Porto, Portugal

**Keywords:** Pheochromocytoma, Catecholamines, Adrenalectomy, Laparoscopy, Complications

## Abstract

**Purpose:**

Pheochromocytoma is a rare neuroendocrine tumor. Despite the low incidence, these tumors are of indisputable importance. This study aimed to analyze the management of pheochromocytoma in a referral center, with an emphasis on the minimally invasive adrenalectomy, which is the preferred therapeutic approach.

**Methods:**

A retrospective analysis was performed on a cohort of patients diagnosed with pheochromocytoma who underwent adrenalectomy between January 2013 and December 2022. Clinical data including demographics, timelines, symptomatology, comorbidities, biochemical markers, genetic testing, surgical details, and follow-up outcomes, were collected and analyzed.

**Results:**

The cohort included 44 patients, predominantly women (52.27%), with a median age of 53.39 years (range 13–83). Most of patients exhibited paroxysmal symptoms suggesting catecholamine excess. Documented hypertension was the most frequent (86.36%), along with glucose anomalies (40.01%) and anxiety disorder (31.82%). Genetic testing was performed in 36 (81.81%) patients and 14 (38.88%) revealed a positive result, predominantly *RET* pathogenic variant. Laparoscopic surgery was performed in 34 (79.07%) patients, showing significantly shorter operative time (2.5 h vs. 4.25 h, t-test p < 0,001) and fewer complications (23.53% vs 77.78%, p = 0.008). Postoperative complications occurred in 36.36% of the patients, mostly mild (grade I, 56.25%), with no mortality. *SDHB* pathogenic variant correlated with both recurrent and metastatic disease (p = 0.006). One-year follow-up reported 9.09% recurrence and 6.82% metastasis.

**Conclusions:**

Adrenalectomy demonstrated a high safety and effectiveness. This study exhibited a higher rate of genetic testing referral than other studies. Despite past advances, there is still a need for further studies to establish protocols and evaluate new techniques.

## Introduction

Pheochromocytoma is a rare neuroendocrine tumor derived from chromaffin cells in the adrenal medulla, characterized by the ability to secrete catecholamines: epinephrine, norepinephrine, and dopamine [1].

The estimated incidence of the tumor varies between 2–8 per million cases per year, but this increases from 0.2% to 0.6% in the hypertensive population [2, 3]. Comparing with studies from the past, it is observed that there is currently an increase in the incidence of this tumor, primarily due to the improvement of noninvasive imaging modalities in clinical practice [4, 5].

The variability of symptoms depends on the type, amount, and pattern of catecholamine secretion, which can be challenging for diagnosis. The presentation spectrum includes the classic triad of symptoms: palpitations, headaches, and diaphoresis [6, 7]. Due to hypersecretion of catecholamines, late diagnosis may lead to fatal cardiac events [8, 9].

Pheochromocytomas have a strong association with genetic inheritance and are linked to specific syndromes, including neurofibromatosis type 1, von Hippel–Lindau (VHL) syndrome, and multiple endocrine neoplasia type 2 (MEN2) [10, 11]. About 40% of patients have pathogenic variants in susceptibility genes, which may have implications not only in symptomatology but also in frequency of metastatic or recurrent disease [12].

The current clinical guidelines for the diagnosis recommend either total fractionated metanephrines or plasma-free metanephrines as an initial screening [13, 14]. Once there is biochemical evidence, subsequent imaging and functional studies should take place [10].

Surgical resection is the only curative treatment, with laparoscopy as the preferred approach. Due to hemodynamic liability, this procedure is still challenging [6, 15].

Overall, the surgical outcomes for management of pheochromocytoma exhibit high rate of success, with low intraoperative mortality and low recurrence of the tumor [16]. Nevertheless, despite the limited number of clinical studies and the recent advances in diagnostic methods, genetic tests, and surgical procedures, the validity of correlations remains a subject of uncertainty.

This study aimed to analyze the experience of pheochromocytoma treatment in a Portuguese referral center.

## Methods

This retrospective cohort study was conducted at the General Surgery Department of *Centro Hospitalar Universitário São João* (CHUSJ), Portugal. All patients with an International Classification of Diseases version 10 (ICD-10) code of 0GB24ZZ (adrenalectomy), that underwent surgical treatment between 1 January 2013 and 31 December 2022, with pheochromocytoma confirmed by histopathological analysis were eligible for inclusion. All data was collected from the digital medical records and was reviewed manually. Those without clear histological confirmation of pheochromocytoma post tumor resection were excluded.

For each patient, the following data was gathered: age at the diagnosis, dates of diagnostical procedures, dates of curative procedures, gender, presenting symptoms, comorbidities, genetic testing for pathogenic variants, familiar history, imaging, side of the tumor, type of procedure, operative time, number and description of postoperative complications, preoperative treatment, presence of extra-adrenal disease, occurrence of metastasis, chemotherapy, and radiotherapy treatment. Baseline hormonal measurements (24 h urinary epinephrine, norepinephrine, and dopamine, total metanephrines, fractionated epinephrine and normetanephrine, and plasma metanephrines and catecholamines) were performed. Local-regional recurrence was defined as the recurrence of the tumor after surgery and normalization of the biochemical tests.

The present work follows the Strengthening the Reporting Of Cohort Studies in Surgery (STROCSS) criteria. The Ethics Committee of the CSHUJ approved the presented observational retrospective study (No 219/2023).

Data analysis was performed using RStudio software. A two-sided P value of <0.05 was considered statistically significant.

## Results

### Patients

Of the 44 patients identified, 23 (52.27%) were women, with a median age at diagnosis of 53.39 years (range 13–83). The median time between diagnosis and surgery was 44.80 days (range 6–159). Baseline characteristics of the patients are shown in Table 1.

**Table 1:** Baseline characteristics of the patients

	n (%)
Sex	
Male	21 (47.73)
Female	23 (52.27)
Age at time of surgery, median (range)	53.39 (13–83)
Days between diagnosis and surgery, median (range)	44.80 (6–159)
Clinical presentation at diagnosis	
Asymptomatic	9 (20.45)
Headaches	21 (47.73)
Palpitations	21 (47.73)
Diaphoresis	17 (38.64)
Classic triad	13 (29.55)
Nausea	14 (31.82)
Thoracic pain	13 (29.55)
Abdominal pain	10 (23.26)
Comorbidities	
Hypertension	38 (86.36)
Hypertensive crisis	18 (40.91)
Glucose anomalies	18 (40.91)
Anxiety	14 (31.82)
Cardiomyopathy	15 (34.09)
Takotsubo syndrome	5 (11.36)
Heredity	
Familiar history	6 (13.63)
Positive genetic tests	14 (38.88)

Of all patients, only 9 (20.45%) presented as asymptomatic. The majority of patients had documented paroxysmal symptoms suggestive of catecholamine excess before diagnosis, with predominance of the classic symptoms: headache (n = 21; 47.73%), palpitation (n = 21; 47.73%) and diaphoresis (n = 17; 38.64%). Thirteen (29.55%) patients presented with the classic triad. Additional symptoms were evaluated: nausea was present in 14 (31.82%) patients, thoracic pain in 13 (29.55%) patients and abdominal pain in 10 (23.26%) patients. Five (11.36%) patients presented with cardiogenic shock, 1 man and 4 women.

Out of all patients, 38 (86.36%) had documented some form of hypertension, and 18 (40.91%) presented hypertensive crises at some point before final diagnosis. Glucose anomalies were observed in 18 (40.91%) patients and anxiety disorder in 14 (31.82%) patients. Fifteen (34.09%) patients had cardiomyopathy documented, 5 of which presenting with Takotsubo syndrome. Only 7 (15.91%) patients had smoking history.

Genetic testing was performed in 36 (81.81%) patients and 14 (38.88%) had a disease-causing germline pathogenic variant. Of all the positive results, pathogenic variants in *RET* gene was the most common, present in more than a quarter (n = 4; 28.57%) of the patients. Pathogenic variants were also found in the *SDHB* gene in 3 (21.4%) patients. Pathogenic variants in the following 5 genes - *NF1*, *VHL*, *SDHD*, *MSH2*, and *TSC2* - were found in 1 (7.14%) patient each. The other 2 (14.28%) patients had a positive result for variants of uncertain significance.

Only 6 (13.63%) patients had previous familiar history of pheochromocytoma, with these being younger than patients without familiar history (mean age 32.8 vs. 56.63, t-test p = 0.005).

### Biochemical tests and radiology

The main analytical findings are given in Table 2.

**Table 2:** Analytical findings of the patients before surgery

Analyte	n (%)	Median value	IQR
Urine			
Metanephrine	37 (84.09)	1054.45	157.65–22260.21
Normetanephrine	38 (86.36)	971.69	587.02–2529.80
Epinephrine	29 (65.91)	21.45	3.00–37.85
Norepinephrine	33 (75.00)	49.23	33.05–452.98
Dopamine	33 (75.00)	209.92	125.41–286.17
Plasma			
Metanephrine	4 (9.09)	888.00	748.00–950.80
Normetanephrine	5 (11.36)	2184.00	885.00–2186.00
Epinephrine	11 (25.00)	137.00	42.00–289.00
Norepinephrine	11 (25.00)	927.00	547.00–1483.00
Dopamine	3 (6.82)	45.93	31.90–74.00

Most of the patients had determination of 24 h urinary fractionated metanephrine and normetanephrine, which were measured in 37 (84.09%) and 38 (86.36%) patients, respectively. Out of those, 24 (64.86%) patients demonstrated biochemical evidence of urinary metanephrine excess, and 33 (86.84%) patients demonstrated biochemical evidence of urinary normetanephrine excess. The median increase was 3.5 and 2.7 times above upper normal laboratory range for the urinary metanephrines and urinary normetanephrine, respectively.

In all patients, computerized tomography (CT) scan or magnetic resonance imaging (MRI) were performed. Additional functional imaging with ^123^I-metaiodobenzylguanidine (MIBG) scintigraphy scan was done in 37 (84.09%) patients and ^18^F-positron emission tomography (PET) scan was performed in 18 (40.91%) patients.

Forty (90.91%) patients had CT scan documented. Out of these, 17 (42.50%) presented as heterogeneous masses with central areas of necrosis and cystic change, 19 (47.50%) presented as solid and heterogeneous masses with density higher than 20 Hounsfield unit (HU), and 4 (10%) presented as heterogeneous masses with density lower than 10 HU, suggesting an adenoma. Of these 4 patients, 1 had biochemical evidence of urinary metanephrines excess, and 3 had radiological evidence in I-123 MIBG scintigraphy scan, changing the final diagnosis to pheochromocytoma. Seventeen (38.63%) patients had MRI documented. Out of these, 15 (88.24%) presented as slightly hypointense in T1 and markedly hyperintense in T2, and 2 (11.76%) presented as a hyperintense signal in T1 and T2. Of these 2, 1 had biochemical evidence of urinary metanephrines excess, and 1 had radiological evidence in I-123 MIBG scintigraphy scan, supporting the diagnosis of pheochromocytoma.

### Surgical outcomes

The surgical outcomes are shown in Table 3.

**Table 3:** Surgical Outcomes

	n (%)
Tumor location	
Right side	24 (54.55)
Left side	18 (40.91)
Bilateral	2 (4.55)
Pre-operative α-blockade	41 (93.18)
Type of adrenalectomy	
Laparoscopic	34 (79.07)
Open surgery	9 (20.93)
Extra-adrenal disease	8 (18.18)
Recurrent disease	4 (9.09)
Metastatic disease	3 (6.82)
Post-surgical complications	
Complications 24 h after surgery	16 (36.36)
Grade I^a^	9 (56.25)
Grade II^a^	5 (31.25)
Grade III^a^	1 (6.25)
Grade IVa^a^	1 (6.25)
Complications 1 month after surgery	2 (4.55)
Complications 1 year after surgery	0 (0)

^a^Relative percentage of the patients who had complications.

Almost every patient (n = 41; 93.18%) were treated with α-adrenergic blockade prior to surgery with phenoxybenzamine. Among the three patients who did not undergo α-adrenergic blockade, two of them were due to the absence of symptoms that did not suggest the presence of the tumor, while the third lacked a specific explanation for the absence of α-adrenergic blockade.

Out of 44 adrenalectomies, 24 (54.55%) were in the right side, 18 (40.91%) in the left side, and 2 (4.55%) bilateral. Bilateral disease was significantly associated with *RET* pathogenic variant (Chi-Squared test p = 0.003) (Table 4).

**Table 4:** Correlation between genetic pathogenic variants and bilateral disease

	Bilateral disease (p-value)
Positive genetic tests (pathogenic variants)	0.281
*RET*	0.003^a^
*SDHB*	1
*NF1*	1
*VHL*	1
*SDHD*	1
*MSH2*	1
*TSC2*	1

^a^Significance value (p < 0.05)

Laparoscopic surgical approach was performed in 34 (79.07%) patients and open surgery was used to treat 9 (20.93%) tumors. Among these 9, 4 were due to the large size of the tumor (exceeding 6 cm), 3 were attributed to adhesions from prior surgical interventions and 2 were related to bilateral disease.

The median time of surgery was shorter for the laparoscopy when compared with the open surgery (2.5 h vs. 4.25 h, t-test p < 0.001). Time of surgery in asymptomatic patients was significantly shorter than in symptomatic patients (2 h vs. 3 h, t-test p = 0.027).

Clavien–Dindo Scale was used for classification of surgical complications. Complications in the first 24 h after surgery were documented in 16 (36.36%) patients: grade I were recorded in 9 (56.25%) patients (most of the patients with mild pain symptoms and one patient with elevation of inflammatory markers, without need of intervention), grade II in 5 (31.25%) patients (three patients with hypotension, one patient with refractory hypertension and one patient with iatrogenic lesion of the right suprarenal artery, showing signs of right kidney ischemia), grade III in 1 (6.25%) patient (with abdominal fluid collections drainage) and grade IVa in 1 (6.25%) patient (who was admitted into intensive care unit due to heart failure) (Table 3). No patients died from complications of adrenalectomy. Only 2 (4.55%) patients had complications 1 month after the surgery and no complications were found 1 year after the surgery, even in the patient admitted to the intensive care unit regarding surveillance in the postoperative period.

Patients who underwent open surgery were more likely to develop complications 24 h after surgery, than patients in whom laparoscopic approach was performed (78.78% vs 23.53%, Chi-squared p = 0.008) (Table 5).

**Table 5:** Correlation between surgical approach and complications 24 h after surgery

	Total of patients	Complications 24 h after surgery
Laparoscopy	34	8 (23.53%)
Open Surgery	9	7 (77.78%)

A shorter time of surgery was associated with less complications 24 h after surgery (t-test p < 0.001).

There were no statistically significant differences in the development of complications when comparing by different symptoms. Correlations between specific pathogenic variants and complications after surgery were studied, with no association being found (Table 6).

**Table 6:** Correlation between genetic pathogenic variants and surgical complications

	Complications 24 h after surgery (p-value)	Complications 1 month after surgery (p-value)
Positive genetic tests (pathogenic variants)	0.081	0.281
*RET*	0.951	1
*SDHB*	0.601	0.380
*NF1*	1	1
*VHL*	0.769	1
*SDHD*	1	1
*MSH2*	0.769	1
*TSC2*	0.769	1

During the one year follow-up, one patient died due to non-correlated disease.

Extra-adrenal disease was found in 8 (18.18%) patients and it was significantly associated with positive genetic tests (n = 7; 50%; p = 0.05), especially *RET* pathogenic variants (n = 3; 75%; p = 0.039) (Table 7).

**Table 7:** Correlation between genetic pathogenic variants and recurrent disease, metastatic disease and extra-adrenal disease

	Recurrent disease (p-value)	Metastatic disease (p-value)	Extra-adrenal disease (p-value)
Positive genetic tests (pathogenic variants)	0.099	0.680	0.005^a^
*RET*	1	1	0.039^a^
*SDHB*	0.006^a^	0.006^a^	1
*NF1*	0.126	1	1
*VHL*	1	1	0.498
*SDHD*	1	1	1
*MSH2*	1	1	1
*TSC2*	1	1	0.498

^a^Significance value (p < 0.05)

Recurrent disease was reported in 4 (9.09%) patients, three of them at 3 months after surgery, and one at 5 months after surgery, all of them with complete resection of the primary tumor. The recurrence occurred in the contralateral side. Three of these patients were identified with genetics syndromes.

Metastatic disease was found in 3 (6.82%) patients: one at the time of diagnosis and the other two with metastases developed at 2 and 4 years after surgical removal of the primary tumor. All these patients were treated with chemotherapy. The 2 patients that developed metastasis later were additionally treated with ^131^I-MIBG radionuclide therapy and radiotherapy 20 Gy.

Both recurrent and metastatic disease were significantly associated with *SDHB* pathogenic variants (p = 0.006) (Table 7).

## Discussion

Our study highlights the diverse spectrum of clinical presentations of pheochromocytoma, emphasizing the diagnostic challenge for clinicians due to the wide variation of symptoms. It is essential to stress the importance of early detection due to high cardiovascular morbidity and mortality, if not treated.

The findings of this cohort match what other studies have found: pheochromocytoma has no sex predilection and it’s typically diagnosed in the first half of the sixth decade of life [17, 18]. As reported in other studies, the mean age at diagnosis in patients with familiar history was lower than in patients without familiar history of the disease [19].

As documented in other studies, most of the patients had symptoms related to the catecholamine excess [5, 18]. Although the high sensibility and specificity of the classic triad, less than 30% of the patients included presented it [20].

As seen in previous research, in our study, hypertension remained as the hallmark clinical sign of pheochromocytoma [21, 22].

Anxiety is also a frequently present comorbidity among patients with pheochromocytoma. In one hand, this can be explained by the presence of events that can mimic panic attacks, with a sensation of dyspnea, palpitations and headaches. On the other hand, hypersecretion of catecholamines can cause disruption of the medial frontal cortical regions and the amygdala, which are the basis of anxiety and depression [23, 24]. In this study, the proportion of patients diagnosed with anxiety was around 30%, which is consistent with what previous studies have reported [21, 23].

Glycemic disturbances in patients with pheochromocytoma have been a subject of study in the last few years. We observed glucose alterations in 40% of our patients, which corresponds well to the published data [25, 26]. These alterations may be related to the catecholamine-induced insulin resistance or insulin suppression.

The extra secretion of catecholamines leads to impairment of the cardiomyocyte function. That explains why pheochromocytomas are frequently associated with cardiomyopathies, as reported in this cohort [21]. The stress induced by catecholamines excess can also be a trigger for Takotsubo syndrome [27], which was recorded in 5 of 44 patients (11%). Furthermore, the data on this matter is limited, so more research should be done.

As reported in literature, pheochromocytoma crisis with cardiogenic shock occurred at a higher rate among women than men [8].

This series demonstrated higher genetic testing referral rates (81.81%) than in previous studies, which highlights the importance of genetic characterization for clinical management and counseling [22, 28]. Similarly to other studies, we found germline pathogenic variants predominantly linked to MEN2A (*RET* pathogenic variants) [29, 30]. However, in this study, hereditary paraganglioma-pheochromocytoma (PGL/PCC) syndromes (*SDHx* pathogenic variants) were found more frequently than neurofibromatosis type 1 and Von Hippel-Lindau disease. Some studies show that *VHL* is the most common pathogenic variant [18, 28]. This might be related to the fact that these studies had multiple patients who were diagnosed before 20 years old, contrary to this study, that had just 2 patients under 20 years old.

In this cohort, one patient presented with Lynch syndrome, which is a non-classic association with only a few cases reported in the literature [31, 32]. This suggests that more studies should be done in order to understand the association between these two tumors.

Quantification of 24 h urinary fractionated metanephrines demonstrate high sensitivity, affirming their diagnostic value [13, 14]. After the biochemical diagnosis, abdominal CT scan emerges as the cost-effective imaging modality of choice, because of its excellent sensitivity and specificity [3, 29]. In this study, CT scan was the most frequently imaging exam performed (n = 40; 90.91%). Although the radiologic results are variable, the main features identified in this study are congruent with previous studies [10].

Phenoxybenzamine efficacy as the α-adrenergic blockade therapy was demonstrated, leading to perioperative blood pressure control and overall safety [33, 34].

In this study, the majority of unilateral pheochromocytomas were located in the right side. These data are consistent with findings from observational studies done in other hospitals [35, 36].

While bilateral disease occurrence was rare (n = 2; 4.55%), it strongly correlated with *RET* pathogenic variants. However, because of the small sample of patients with bilateral disease, this association should be analyzed carefully. Tumor recurrence was observed in 4 patients, 3 of them with genetic pathogenic variants. These association between genetic background and bilateral disease was congruent with previous studies [18, 36].

The treatment of choice for pheochromocytoma is surgical resection [1, 3]. Laparoscopic surgery has been accepted as the primary surgical procedure. In this study, it was performed in 79% of patients, showing better results regarding both the time of surgery and the rate of complications. These results show that this approach is a safe and feasible option for pheochromocytoma treatment, which is consistent with the literature [33, 37].

The correlation values observed between specific pathogenic variants and post-surgical complications suggest the need for further extensive studies with larger samples, to better understand possible correlations.

This study demonstrated that patients with pheochromocytoma and concomitant *RET* pathogenic variant are at greater risk of developing extra-adrenal forms of the disease. Therefore, when evaluating patients with this variant, this should be considered.

As described in the literature, the prevalence of metastatic disease usually varies between 10–20% [29]. This study identified only 3 patients with metastatic disease, all of them exhibiting related genetic pathogenic variants.

This study showed a strong association between *SDHB* pathogenic variant and the recurrent and metastatic forms of the disease, which is consistent with other studies [38, 39]. These associations highlight the importance of genetic testing in these patients, in order to individualize the therapeutic approach and further genetic counseling that should be extended to the family [11, 40].

We acknowledge some limitations of this study, including its retrospective nature, which can lead to lack of uniformity in the medical records, and considering the rare incidence of this tumor, the relatively small sample size.

A strength of this study lies in the fact that the hospital where it was conducted is a tertiary care referral center for treating pheochromocytoma in Portugal. This is important given the rarity of pheochromocytoma, as this referral center routinely attends the great majority of patients within a vast geographic area. Additionally, this database has a good sample of patients with complete medical records.

In conclusion, these findings underscore the importance of early detection of pheochromocytoma presentation, which can be life-saving. Also, genetic testing has become more common in the management of this tumor, reflecting an improvement in the diagnosis. As expected, this study demonstrates the high safety and effectiveness of the laparoscopy, which showed less complications in the 24 h post-operation period, while maintaining effective resolution of symptoms and low recurrence rate. Despite the advances made over the past decades, there is still a need for further studies to establish protocols and evaluate new adrenalectomy techniques.
